# Draft Genome Sequences of Two *Bacillus anthracis* Strains from Etosha National Park, Namibia

**DOI:** 10.1128/genomeA.00861-16

**Published:** 2016-08-25

**Authors:** Karoline Valseth, Camilla L. Nesbø, W. Ryan Easterday, Wendy C. Turner, Jaran S. Olsen, Nils C. Stenseth, Thomas H. A. Haverkamp

**Affiliations:** a Centre for Ecological and Evolutionary Synthesis (CEES), Department of Biosciences, University of Oslo, Blindern, Oslo, Norway; b Norwegian Defence Research Establishment, Kjeller, Norway; c Department of Biological Sciences, University of Alberta, Edmonton, Alberta, Canada; d State University of New York, Albany, New York, USA

## Abstract

*Bacillus anthracis* strains K1 and K2 were isolated from two plains zebra anthrax carcasses in Etosha National Park, Namibia. These are draft genomes obtained by Illumina MiSeq sequencing of isolates collected from culture of blood-soaked soil from each carcass.

## GENOME ANNOUNCEMENT

*Bacillus anthracis* is a Gram-positive, rod-shaped bacterium which sporulates to survive the transmission between hosts (1). It belongs to the genetically similar *B. cereus* group, and is the causative agent of anthrax (2). Anthrax is endemic to Etosha National Park, Namibia, which has yearly outbreaks of the mortal infection arising from spore-contaminated soil of carcass sites from previous years (3).

Two *B. anthracis* isolates were obtained by culturing serial dilutions on polymyxin-lysozyme-EDTA-thallous acetate (PLET) agar culture medium following previously described protocols (4, 5) from contaminated soil beneath two plains zebra (*Equus quagga*) carcasses (Ca1 and Ca2) (located on 3 March 2014—coordinates, Ca1: S19.031/E015.548; Ca2: S19.037/E015.553). One colony per carcass was picked from the PLET plates and regrown as a lawn on a fresh PLET plate, then colonies were scraped of the PLET agar with a spatula and dispensed in 300 µL phosphate buffered saline (PBS) buffer (1 tablet from Sigma-Aldrich, USA, dissolved in 200 mL dH_2_O, autoclaved at 121°C for 20 min) before isolation. DNA isolation was performed using a FastDNA spin kit for soil (MP Biomedicals, CA, USA) following the manufacturer’s protocol with these alterations: tubes were homogenized with a MiniBead Beater, model 607 (BioSpec Products, OK, USA) for 80 s at 3,450 oscillations/min, before being centrifuged at 14,000 rpm (rpm) for 10 min. Six hundred fifty  µL of the supernatant was removed in step 10 in protocol. In step 13 of the protocol, spin filters were centrifuged for 3 min without emptying the catch tube. DES buffer was heated to 55°C in Dri-Bath, type 17600 (Thermolyn, IA, USA) before addition. Then, tubes were put on a heat block for 5 min at 55°C. Finally, the DNA extracts were sterilized using an Ultrafree Durapore PVDF 0.1 µM spin filter (Millipore, Darmstadt, Germany) by centrifugation at 11,000 rpm for 3 min.

The bacterial DNA isolates were whole-genome sequenced at the Norwegian Sequencing Centre (NSC) using the TruSeq Nano reagents (Illumina, Inc., San Diego, CA, USA) and Illumina MiSeq with the following parameters: paired-end, insert size of 500 bp and a read length of 300 bp. Sequences were cleaned with AdapterRemoval 1.5.4 (6) and assembled in CLC workbench v8 using the AdapterRemoval 1.5.4 output files for singleton, pair1, and pair2. The genomes were annotated using the NCBI PGAP pipeline. The sequenced *B. anthracis* isolates from Ca1 and Ca2 were named K1 and K2, respectively, after the Norwegian word for carcass, “kadaver.” The K1 draft genome consisted of 38 contigs covering of 5,456,561 bp, with a G+C content of 35.1%, 5,837 genes, and 5,549 coding sequences (CDSs). The K2 draft genome is very similar with 38 contigs covering 5,461,141 bp, G+C content of 35.1%, 5,856 genes, and 5,549 CDSs.

### Accession number(s).

Both whole-genome shotgun projects have been deposited in DDBJ/ENA/GenBank under the accession no. LBBZ00000000 (K1) and LBCA00000000 (K2).
